# Long noncoding intronic RNAs are differentially expressed in primary and metastatic pancreatic cancer

**DOI:** 10.1186/1476-4598-10-141

**Published:** 2011-11-13

**Authors:** Ana C Tahira, Márcia S Kubrusly, Michele F Faria, Bianca Dazzani, Rogério S Fonseca, Vinicius Maracaja-Coutinho, Sergio Verjovski-Almeida, Marcel CC Machado, Eduardo M Reis

**Affiliations:** 1 Departamento de Bioquímica, Instituto de Química, Universidade de São Paulo, 05508-900, São Paulo, SP, Brasil; 2 Departamento de Gastroenterologia (LIM-37), Faculdade de Medicina, Universidade de São Paulo, 01246-903, São Paulo, SP, Brasil

**Keywords:** pancreatic cancer, molecular markers, noncoding RNAs, intronic transcription, metastasis, *MAPK *, pathway, cDNA microarrays

## Abstract

**Background:**

Pancreatic ductal adenocarcinoma (PDAC) is known by its aggressiveness and lack of effective therapeutic options. Thus, improvement in current knowledge of molecular changes associated with pancreatic cancer is urgently needed to explore novel venues of diagnostics and treatment of this dismal disease. While there is mounting evidence that long noncoding RNAs (lncRNAs) transcribed from intronic and intergenic regions of the human genome may play different roles in the regulation of gene expression in normal and cancer cells, their expression pattern and biological relevance in pancreatic cancer is currently unknown. In the present work we investigated the relative abundance of a collection of lncRNAs in patients' pancreatic tissue samples aiming at identifying gene expression profiles correlated to pancreatic cancer and metastasis.

**Methods:**

Custom 3,355-element spotted cDNA microarray interrogating protein-coding genes and putative lncRNA were used to obtain expression profiles from 38 clinical samples of tumor and non-tumor pancreatic tissues. Bioinformatics analyses were performed to characterize structure and conservation of lncRNAs expressed in pancreatic tissues, as well as to identify expression signatures correlated to tissue histology. Strand-specific reverse transcription followed by PCR and qRT-PCR were employed to determine strandedness of lncRNAs and to validate microarray results, respectively.

**Results:**

We show that subsets of intronic/intergenic lncRNAs are expressed across tumor and non-tumor pancreatic tissue samples. Enrichment of promoter-associated chromatin marks and over-representation of conserved DNA elements and stable secondary structure predictions suggest that these transcripts are generated from independent transcriptional units and that at least a fraction is under evolutionary selection, and thus potentially functional.

Statistically significant expression signatures comprising protein-coding mRNAs and lncRNAs that correlate to PDAC or to pancreatic cancer metastasis were identified. Interestingly, *loci *harboring intronic lncRNAs differentially expressed in PDAC metastases were enriched in genes associated to the MAPK pathway. Orientation-specific RT-PCR documented that intronic transcripts are expressed in sense, antisense or both orientations relative to protein-coding mRNAs. Differential expression of a subset of intronic lncRNAs (*PPP3CB*, *MAP3K14 *and *DAPK1 loci*) in metastatic samples was confirmed by Real-Time PCR.

**Conclusion:**

Our findings reveal sets of intronic lncRNAs expressed in pancreatic tissues whose abundance is correlated to PDAC or metastasis, thus pointing to the potential relevance of this class of transcripts in biological processes related to malignant transformation and metastasis in pancreatic cancer.

## Background

Pancreatic ductal adenocarcinoma (PDAC) is the most common pancreatic neoplasm and accounts for > 85% of pancreatic tumor cases [1]. PDAC is a devastating disease with very poor prognosis for which the only curative treatment is resection surgery [2]. However, only 15-20% of patients have resectable pancreatic tumor, and from these only 20% presents a 5-year survival, which results in an average 5-year survival rate of 3-5% [1]. PDAC aggressiveness is mainly associated to the lack of early diagnosis tools and the limited response to available treatments [2].

Large-scale gene expression studies of tumor samples have been extensively employed to delineate the molecular pathways and cellular processes involved in tumorigenesis and progression of PDAC [3] and to search for novel biomarkers for diagnosis and molecular targets for therapeutic intervention in pancreatic cancer [4]. In spite of the wealth of information generated in recent years on the most frequent molecular alterations found in PDAC [5], there are still important open question in pancreatic cancer biology such as the profound resistance of primary and metastatic PDAC to chemo- and radiotherapy [6]. Regarding the identification of molecular markers for pancreatic cancer diagnostic/prognostic, while some promising candidate genes have been proposed [4], none have been proven effective to significantly improve early detection and to reduce mortality/morbidity of the disease. Thus, a better understanding of the molecular basis of pancreatic cancer is required for the identification of more effective diagnostic markers and therapeutic targets.

Over the last decade, advances in genome-wide analyses of the eukaryotic transcriptome have revealed that the majority of the human genome is transcribed, producing large numbers of long (> 200 nt) noncoding RNAs (lncRNAs) mapping to intronic and intergenic regions [7-10]. These include subsets of polyadenylated and non-adenylated transcripts that accumulate differently in the nucleus and cytoplasm of cells [10,11]. While only a small fraction of lncRNAs have been characterized in detail, it is clear that these transcripts may act through diverse molecular mechanisms and play regulatory and structural roles in important biological processes, such as in genomic imprinting, chromosome inactivation, cell differentiation and development, cell proliferation, protein nuclear import, organization of nuclear domains and apoptosis (see [12] for a review).

Altered expression of lncRNAs has been documented in different types of human cancer [13-15] prompting an increasing interest in their use as biomarkers for diagnosis and prognosis as well as potential therapeutic targets [14,16-19]. Increased expression of the lncRNA *MALAT-1 *has been observed in several types of tumors, including metastatic non-small cell lung cancer [19]. Recently, augmented levels of *HOTAIR *in primary breast tumors were shown to correlate with breast cancer invasiveness and metastasis [18]. Measurement of lncRNA *PCA3 *in patient urine samples has been shown to allow more sensitive and specific diagnosis of prostate cancer than the widely used marker prostate-specific antigen (PSA) [16]. The lncRNA *HULC *is highly expressed in hepatocarcinoma patients and detected in the blood by conventional PCR methods [20].

There are several reports of aberrant expression of microRNAs in PDAC [21,22], and there is potential in their use as biomarkers for disease diagnosis [23,24]. However, there is a paucity of information regarding the expression of lncRNAs in pancreatic cancer. In an interesting study performed by Ting et al. it was observed the aberrant overexpression of satellite repeat RNAs (HSATII) ranging from 100 to 5000 nt in patients with PDAC [25]. Interestingly, detection of HSATII by RNA in situ hybridization was able to correctly diagnose PDAC in tumor biopsies, including cases in which the histopathology was non-diagnostic [25].

Our group has previously shown that most (at least 74%) annotated protein-coding gene *loci *generate intragenic lncRNAs that map to intronic regions [26]. Possible relevance of intronic lncRNAs to neoplastic processes was proposed following the observation that subsets of these transcripts are present in gene expression signatures correlated to the degree of malignancy in prostate cancer [17] or to tissue histology in head and neck tumors [27] and renal cell carcinoma [28]. In addition, a number of intronic lncRNAs were found to be regulated by androgen stimulation of cultured prostate cancer cells [29], indicating that these transcripts are expressed in a regulated manner and thus, corroborating the idea that intronic lncRNAs are biologically relevant.

In this study, we used a custom cDNA microarray platform with probes for lncRNAs expressed from intronic and intergenic regions of the human genome, as well as for a selected set of cancer-related protein-coding genes to generate expression profiles from a collection of tumor and non-tumor pancreatic tissue samples. Expression of intronic/intergenic lncRNAs subsets was detected across all samples tested. Enrichment of promoter-associated chromatin marks indicate that these transcripts originate from independent transcriptional units. Over-representation of conserved DNA elements and stable secondary structure predictions suggest that at least a fraction of these transcripts are under evolutionary selection and thus potentially functional.

Importantly, we identified expression signatures comprising long noncoding RNAs that are significantly correlated with primary and metastatic ductal pancreatic adenocarcinoma. This suggests that lncRNAs are modulated during tumorigenesis and tumor progression and therefore may participate in molecular processes relevant to malignant transformation and metastasis in pancreatic cancer.

## Results

### Long noncoding RNAs from intronic and intergenic regions are expressed in neoplastic and non-tumor pancreatic tissues

In this work, a custom spotted cDNA microarray with approximately 4,000 elements was used to investigate the expression patterns of a collection of protein-coding transcripts and putative noncoding RNAs in clinical samples of primary and metastatic tumor, chronic pancreatitis and histologically normal pancreatic tissue. This array platform has been described previously [17,28] and contains probes that interrogate 2,371 RefSeq mRNAs from genes associated with cancer in the literature, as well as 984 transcripts mapping to intronic or intergenic regions of the genome and to known lncRNAs. Fluorescent cRNA targets generated from 38 pancreatic tissue samples (15 primary adenocarcinoma, 9 histologically normal adjacent tissue, 6 metastatic samples and 8 chronic pancreatitis) were individually hybridized to microarrays in replicate. After data filtering (see Methods for details), 1,607 transcripts were detected as expressed in at least one histological type, being 1,267 protein-coding mRNAs and 340 putative noncoding RNAs, including transcripts with no overlap to RefSeq exons, i.e, mapping to intronic and intergenic regions. Only candidate lncRNAs sequences that showed genomic alignments with at least 90% identity and coverage were further analyzed, resulting in 335 transcripts (22 known lncRNAs, 240 putative lncRNAs mapped to intronic regions and 73 to intergenic regions).

Expression of comparable fractions of protein-coding mRNAs and putative long noncoding transcripts mapping to intronic and intergenic regions was detected in all histological tissue types (Table 1). The fraction of intronic lncRNAs detected as expressed in the microarray (240/722 = 0.33) is comparable to that of known RefSeq lncRNAs (22/74 = 0.30) and intergenic lncRNAs (73/188 = 0.39), and lower than the fraction of expressed protein-coding mRNAs (1267/2371 = 0.53). The smaller fraction of lncRNAs detected in pancreatic tissues (0.30-0.39) compared to protein-coding mRNAs (0.53) reflects the observation from other studies that noncoding RNAs are generally less abundant and more tissue-specific than protein coding mRNAs [9,26]. In fact, we observed that the lncRNAs detected in pancreatic tissue samples by array hybridization were on average less abundant than protein-coding transcripts (average intensities 24.9 and 31.6, respectively).

**Table 1:** Gene expression detected in the microarrays according to probe type and pancreatic tissue histology

		Detected as expressed in				
			
Type	# probes in the array	NT (n = 9)	T (n = 15)	M (n = 6)	CP (n = 8)	# expressed probes *
protein-coding mRNA	2371	1106	1167	1198	1230	1267
Known lncRNA (RefSeq)	74	20	19	22	18	22
Intronic lncRNA	722	206	202	238	235	240
Intergenic lncRNA	188	68	68	74	77	78

Total	3355	1400	1456	1532	1560	1607

*To be considered as expressed, a probe signal should be detected above the median array intensity value in at least 75% of samples from at least one histological type.

NT, non-tumor pancreatic tissue; T, primary pancreatic adenocarcinoma; M, metastases from primary pancreatic tumors; CP; chronic pancreatitis.

Of the 240 gene loci harboring intronic lncRNAs that were detected in pancreatic tissues, only 62 had array probes interrogating exons of mRNAs from the same *loci*. From these, 31 (50%) were detected only in intronic regions, pointing to a subset of lncRNAs that conceivably are generated by independent intronic transcription rather than pre-mRNA splicing. Thirty one *loci *were detected by both exonic and intronic probes (50%). For each of these *loci*, Pearson correlation between the expression of the lncRNA and mRNA across all pancreatic tissue samples was calculated. Correlations were generally low (-0.5 < r < 0.5 for 27 out 31 *loci*), with 11 *loci *displaying a negative correlation between expression of the intronic lncRNA and the mRNA, and 20 showing a positive correlation.

To obtain further information regarding the correlation between the 240 intronic lncRNAs expressed in pancreatic tissues and the adjacent exons from the same *loci *we analyzed their expression in a set of nine RNAseq libraries [30]. For each *locus *in each library, the number of tags was normalized by RPKM (Reads Per Kilobase of exon model per Million mapped reads). Pearson correlations between each intronic lncRNA and the adjacent upstream/downstream exons were calculated if all elements (intronic lncRNA, upstream and downstream exons) were detected in at least 4 out 9 RNAseq libraries. Seventy five loci (75/240, 31%) satisfied these criteria and were further analyzed. As expected, we found a high correlation between the expression of exons flanking intronic lncRNAs (66/75 with r > 0.5). We found that about a third of exon/intron pairs showed positive correlation of expression (24/75, r > 0.5). The expression of more than half of exon/intron pairs were poorly correlated (46/75, -0.5 < r < 0.5), and a small fraction was negatively correlated (5/75, r < -0.5). The overall low correlation observed between the expression of intronic lncRNAs and adjacent exons suggest that for the most part intronic lncRNAs are processed and accumulate in the cell at rates distinct from mRNAs produced in the same *loci*, arguing against them being simply remainings of splicing lariats.

**Figure 1:**
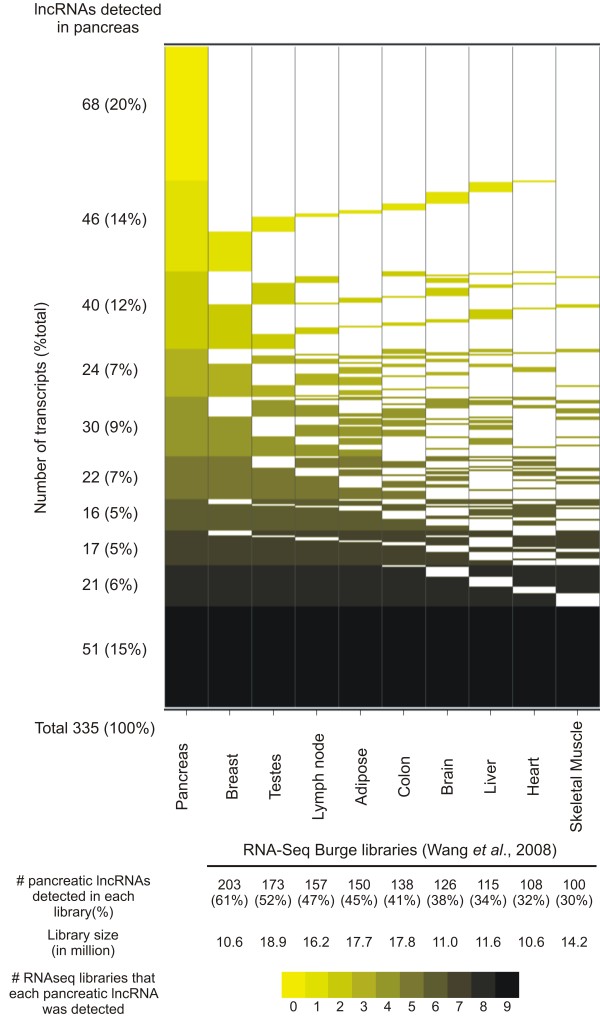
**Intronic and intergenic lncRNAs detected in pancreatic tissues comprise both tissue-specific and broadly expressed transcripts**. Genomic coordinates of 335 intronic/intergenic transcripts detected as expressed in pancreatic tissues were cross-referenced with genomic coordinates of transcripts detected in RNAseq libraries from nine different tissues [30]. LncRNAs were grouped and colored by the number of tissue libraries where their expression was detected (Y axis). RNAseq libraries are ordered in the × axis according to the number of pancreatic lncRNAs detected in each library.

To gain further insight into the expression pattern of the 335 putative lncRNAs, we investigated their expression in other tissue types using publicly available RNAseq datasets generated from nine different tissue histologies [30]. By cross-referencing the genome mapping coordinates of the pancreatic-expressed lncRNAs with coordinates of the RNAseq reads we found that approximately 80% of the former (267/335) are detected in at least one other human tissue (Figure 1).

Coding potential of the 335 sequences mapping to intronic and intergenic regions was investigated using the Coding Potential Calculator (CPC) software [31]. This analysis showed that most sequences (322/335, 96%) have little or no protein coding potential. Thus, we suggest that most of the intronic and intergenic transcripts detected in pancreatic tissues are indeed noncoding RNAs.

To document the length of the intronic transcripts expressed in pancreatic samples we compared the set of intronic RNA sequences (n = 240) with sequences resulting from the assembly of ESTs and mRNAs deposited in GenBank that map to intronic regions of the genome, previously generated in our group and which is available as a UCSC Genome Browser custom annotation track [26]. We found that 190 out 240 intronic transcripts (79%) are represented by an assembled sequence contig. The mean length of the intronic contigs is 779 bp, whereas the individual ESTs have a mean length of 428 bp, suggesting that the ESTs spotted on the microarray are partial sequences of longer noncoding RNA transcripts.

We also investigated the proximity of 73 intergenic lncRNAs to UTRs of annotated genes to evaluate if these transcripts could represent untranslated regions of incomplete mRNAs. We found that 14 intergenic transcripts (14/73, i.e. 19%) map within 1 kb from a known 3'or 5' UTR and could potentially extend the 3' or 5' untranslated region of a known protein-coding mRNA. The remaining 59 transcripts map at least 1 kb away from a known mRNA and possibly constitute yet unannotated intergenic noncoding RNAs.

To investigate if the intronic and intergenic RNAs detected in pancreatic tissue could be precursors of small regulatory ncRNAs, we compared the set of 335 lncRNAs expressed in pancreas to microRNA and snRNA sequence databases [32,33]. No significant similarity to known small RNA was found, except for one sequence that mapped to the *SNOR89 locus*. Considering that the average length of micro RNA precursors (> 1,000 nt) is greater than the average EST length (468 nt) we extended the genomic coordinates of probe sequences by 1 kb at both ends and repeated the sequence comparison with known small RNA datasets. Using this approach, we found four putative extended EST sequences that show high similarity to seven additional small RNAs: *hsa-mir-1259, hsa-mir-326, hsa-mir-4269, hsa-mir-675, SNORD12, SNORD12B *and *SNORD12C*.

Sequence conservation among species is generally viewed as an indication of functional significance of a given genomic feature. We searched for evidence of sequence conservation within the set of intronic/intergenic lncRNAs expressed in pancreatic tissues by comparing their mapping coordinates with those from conserved DNA elements in vertebrates (phastCons 46way vertebrates), mammals (phastCons 46way placental) and primates (phastCons 46way primates) obtained from the UCSC genome browser. After normalization by the number of conserved elements present in each group, relatively greater overlap with conserved DNA elements was observed within primate, mammalian and vertebrate sequences, in this order (Additional File 1, Figure S1). The overlap of intronic/intergenic RNAs with evolutionarily conserved DNA elements was greater than the expected by chance alone, as judge by the overlap attained with a set of randomly selected intronic/intergenic DNA sequences with same length and CG% content (Fisher's exact test *p *< 0.05, Additional File 1, Figure S1). In addition, a fraction of the lncRNAs mapping to intronic/intergenic regions (49 sequences out 335 analyzed, 15%) appear to fold into stable RNA secondary structures (P > 0.5) (http://verjo102.iq.usp.br/sites/tahira/structures.html), as predicted by the RNAz program [34]. Altogether, these observations provide additional support to the notion that at least a fraction of the noncoding transcripts mapping to intronic/intergenic regions may exert functional roles in pancreatic cells.

### Enrichment of promoter-associated chromatin marks (H3K4me3) and start sites of capped transcripts suggest that intronic lncRNAs are independent transcriptional units

Given the paucity of information about the biogenesis of lncRNAs originated from intronic and intergenic regions, we searched for regulatory elements in the genome that could be associated to their transcriptional control. First, we investigated the distribution of trimethylation of lysine 4 in histone 3 (H3K4me3), a chromatin modification associated with regions of transcription initiation [35], in the vicinity of intronic/intergenic lncRNAs. Genomic coordinates of H3K4me3 marks measured in 13 cell lineages [35] were obtained from the UCSC Genome Browser. Only H3K4me3 marks with a *p *< 10^-5 ^were used to limit the experimental noise. The nearest H3K4me4 mark relative to the known boundaries of intronic/intergenic lncRNAs (based on sequenced ESTs) expressed in pancreatic tissues was selected and the distance annotated. As a control, the same analysis was performed using 100 random sets of intronic or intergenic DNA sequences with same length and GC content.

**Figure 2:**
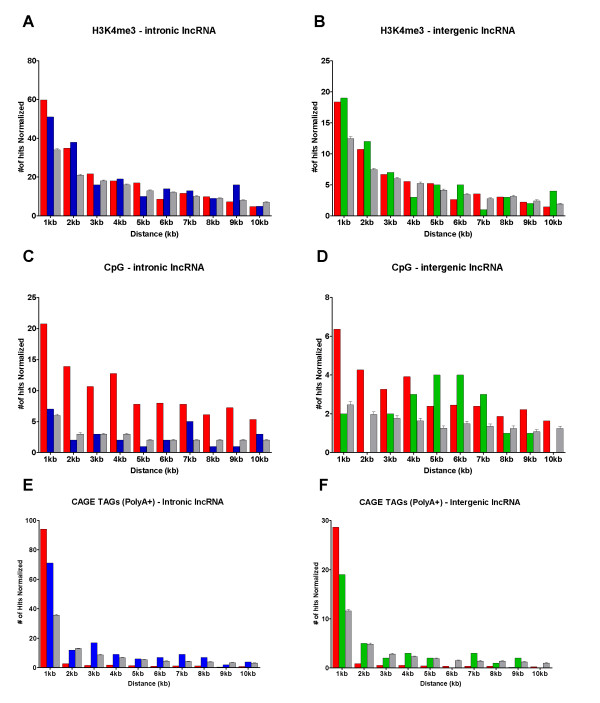
**Genomic *loci *encoding intronic lncRNAs are enriched in promoter-associated histone marks and start sites of capped transcripts**. Distance distribution (X axis) of promoter-associated chromatin marks H3K4me3 binding sites (panels A, B), CpG islands (panels C, D) and CAGE Tags (panels E, F) relative to genomic coordinates of intronic (blue bars) and intergenic (green bars) transcripts expressed in pancreatic tissues (Y axis) were calculated. For comparison, distribution distances were calculated for an equal number of protein-coding mRNAs (red bars) and for randomly selected intronic or intergenic genomic sequences with the same length and % GC of pancreas expressed lncRNAs (light gray bars).

An enrichment of H3K4me3 marks was observed closer to the known boundaries for the set of intronic transcripts expressed in pancreatic tissue samples (Figure 2, panel A, blue bars). The distance distribution of H3K4me3 marks relative to known boundaries of intronic transcripts was significantly different (KS test, highest *p *< 0.05) from that observed for a random set of sequences (same length and %CG), indicating that it is not explained by chance alone. The same analysis was performed with the set of expressed intergenic regions. Although we observed a higher frequency of H3K4me3 marks closer to the known boundaries of the intergenic transcripts, we found no statistically significant difference in their distribution relative to the random control set (Figure 2, panel B, green bars).

As expected, we observed a higher frequency of H3K4me3 marks closer to the known boundaries of protein-coding mRNAs (Figure 2, panel A, red bars). This distribution is statistically different from the one obtained with a control comprising a random sequence set (KS test, highest *p *< 0.01). No statistically significant difference was observed between pancreas-expressed intronic/intergenic lncRNAs and mRNAs regarding the distributions of promoter-associated H3K4me3 marks, indicating that these distributions are similar. The enrichment of promoter-associated H3K4me3 at the vicinity of intronic/intergenic pancreatic-expressed transcripts argues that these transcripts are independent transcriptional units.

We also investigated the distribution of annotated CpG islands relative to EST probes representing protein-coding mRNAs and noncoding intronic/intergenic RNAs expressed in pancreatic tissues. To pursue this analysis we used the genomic coordinates of CpG islands available as a UCSC genome browser track. First, we cross-referenced the coordinates of annotated CpG islands with those of EST probes representing mRNAs expressed in pancreatic tissues, which showed an enrichment towards EST boundary coordinates (Figure 2, panel C, red bars), significantly different from the distribution observed by a random sequence set (KS test, p < 0.001). No statistically significant association with CpG islands was observed for intronic or intergenic sequence sets relative to random sets with same length and CG% content (KS test, *p-value *> 0.05) (Figure 2, panels C and D, blue and green bars).

We also compared the known start sites of intronic/intergenic lncRNAs with CAGE tags generated from poly(A+) RNA from 6 different cell lineages (RIKEN). We note that this set does not include CAGE libraries derived from pancreatic tissues. As pre-processing, coordinates of overlapped tags were clustered and only clusters containing at least 5 tags were considered for further analysis. Next, we calculated the distance of the closest CAGE tag cluster to intronic/intergenic lncRNAs, protein-coding mRNAs, and to random genomic sequences. A significant enrichment (KS test, p < 0.05) of CAGE tags within 1kb of the known start of intronic lncRNAs expressed in pancreatic tissues was observed (Figure 2, panel E, blue bars). Although a higher frequency of CAGE tags closer to the known start site of intergenic lncRNAs was observed, the enrichment was not statistically significant when compared to the random control set (Figure 2, panel F, green and gray bars).

### Identification of a gene expression signature correlated to ductal pancreatic cancer comprising protein-coding and lncRNAs

**Figure 3:**
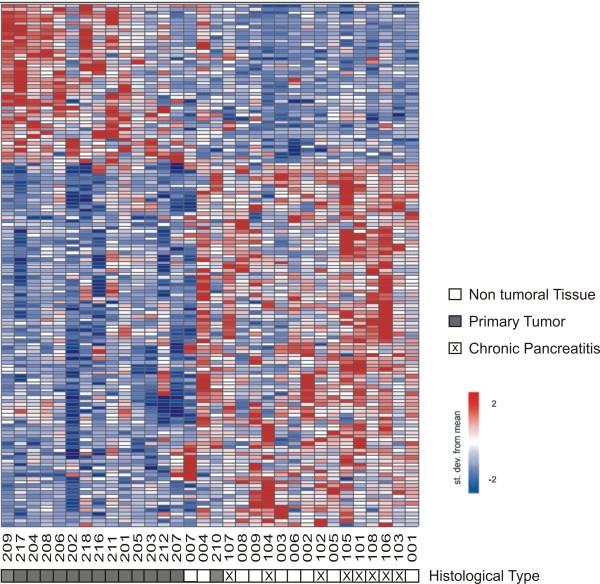
**A gene expression signature of pancreatic adenocarcinoma**. A two-class statistical analysis (see ***Methods***) identified 147 transcripts (rows) differentially expressed (FDR ≤10%) between primary adenocarcinoma samples and histologically normal and chronic pancreatitis samples combined (columns). Patient ID numbers are shown below the columns. Forty three transcripts mapping to intronic or intergenic regions were identified (43/147, i.e. 29%). Expression level of each gene is represented by the number of standard deviations above (red) or below (blue) the average value for that gene across all samples. Samples are ordered according to their individual correlation to the average profile of the primary tumor samples. Sample tissue histology is shown below each patient ID.

To gain further insights on the putative biological relevance of intronic/intergenic noncoding RNAs in pancreatic cancer we investigated their relative expression in tumor and non-tumor pancreatic tissues. Identification of genes specifically deregulated in malignant pancreatic epithelial cells is frequently confounded by an augmented stromal component in the latter due to the presence of proliferating stromal cells and infiltrating inflammatory cells [36,37]. A similar desmoplastic reaction is observed in chronic pancreatitis [38,39]. To favor the identification of genes specifically altered in neoplastic pancreatic cells, we performed a two-class analysis comparing the expression profiles of 15 primary adenocarcinoma samples with nine histologically normal tissue fragments adjacent to tumors combined to eight samples of chronic pancreatitis. Using this approach, we found 147 transcripts differentially expressed in pancreatic tumor samples relative to non-tumor tissues (FDR ≤ 10%). This expression signature comprised 104 protein-coding mRNAs and 43 lncRNAs, being 34 intronic and 9 intergenic transcripts (See Additional file 2, Table S1 for a complete list). As shown in Figure 3, except by one sample (210, primary tumor), the 147-gene signature efficiently discriminated tumor and non-tumor tissues. Conceivably, the prevalence of an inflammatory component in the 210 sample could explain this sample showing an expression profile more similar to chronic pancreatitis samples.

Next, we performed a meta-analysis to compare the list of protein-coding mRNAs presented in the pancreatic tumor expression signature with those identified in other gene expression studies with clinical samples of pancreatic cancer, retrieved from the Pancreatic Expression Database [40] (see Additional file 3, Table S2). Twenty four out 104 protein-coding transcripts detected in our analysis (24/104, i.e. 23%) were reported in at least one of the 12 studies, comprising 15 different analyses, deposited in the Pancreatic Expression Database. From these, expression changes of 17 genes were confirmed by other studies, whereas 3 showed partial agreements and 4 showed an inverted pattern of expression. Confirmed genes included genes already reported in the literature and proposed as biomarkers of pancreatic cancer such as *S100A6*, *TIMP1*, *NF-κB*, *VCL *and *S100P *[5,41-47]. It is worth mentioning that overexpression of *S100P *was detected in 11 different studies (Additional file 3, Table S2). We also detected upregulation of *MBOAT *in pancreatic tumors. Increased expression of MBOAT in ductal pancreatic adenocarcinoma has been already reported and was shown to inversely correlate to patient survival in pancreatic cancer [39].

A gene enrichment analysis using the DAVID analysis suite [48] using as input either the list of protein-coding mRNAs or of intronic transcripts differentially expressed in pancreatic tumors was performed to investigate the over-representation of specific molecular functions, biological processes and cellular components of the Gene Ontology annotation [49]. For this analysis, intronic transcripts were annotated according to the gene *locus *where they map on the genome. Only categories having corrected EASE score < 0.05 [48] were considered as overrepresented.

Among the set of 104 protein-coding mRNAs differentially expressed in pancreatic tumors we found an enrichment of gene categories encoding proteins involved in "focal adhesion" (*p *< 0.03; *TRIP6*, *TRIM25*, *VCL*, *SDC1*, *ARPC2*, *DLC1*, *CDH1*, *ITGB5*), "RNA transport and localization " (*p *< 0.01; *THOC7*, *THOC2*, *RAN*, *NUP85*, *THOC3*) and localizing to "basolateral plasma membrane" (*p *< 0.05; *NOTCH4*, *ARPC1B*, *CDH1*, *TRIP6*, *VCL*, *TRIM25*, *SDC1*, *DLC1*). Deregulation in pancreatic cancer of genes encoding proteins involved in focal adhesion has already been reported in the literature [39]. Noteworthy, the gene category "RNA transport and localization" comprises genes associated to the TREX-complex (*THOC2*, *THOC3*). Increased expression of this complex (Thoc1) in breast cancer correlates with tumor size and the metastatic state of the tumor progression [50], thus suggesting that modulation of the TREX-complex could also have a role in pancreatic cancer. No enriched gene category was found amongst gene *loci *that harbor differentially expressed intronic lncRNAs.

Ingenuity Pathway Analysis (IPA) [51] was used to identify pathways and gene networks represented amongst the sets of protein-coding mRNAs identified in the pancreatic tumor gene expression signature. The most enriched network, "cellular movement, cell-to-cell signaling interactions and endocrine system" (*p *< 10^-43^) comprised 23 differentially expressed transcripts and included most genes represented in the enriched gene categories identified using DAVID (see Additional file 4, Figure S2). Gene networks associated with "cellular movement, skeletal and muscular system development and function and inflammatory response" (17 genes, *p *< 10^-29^) and "carbohydrate metabolism, small molecule biochemistry and infectious disease" (16 genes, *p *< 10^-28^) were also identified.

### Identification of genes correlated to metastasis in pancreatic cancer

**Figure 4:**
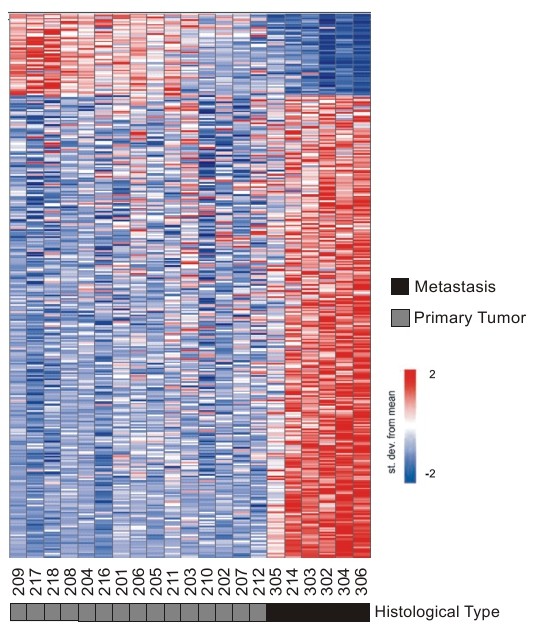
**A gene expression signature correlated to metastasis in pancreatic adenocarcinoma**. Three hundred fifty five transcripts (rows) identified as differentially expressed (FDR ≤ 5%) between metastatic (dark box) and primary tumor (dark gray) samples from 21 patients (columns). Patient ID numbers are shown below the columns. One hundred thirty four intronic and intergenic lncRNAs were identified, comprising 38% of the metastasis signature. Expression level of each transcript is represented by the number of standard deviations above (red) or below (blue) the average value across all samples. Samples are ordered according to their individual correlation to the average profile of primary tumor samples. Tissue histology is shown below each patient ID.

A hallmark of pancreatic cancer is the high prevalence of metastatic disease, whose molecular basis is poorly understood. To search for protein-coding and long noncoding RNAs with expression levels correlated to the metastatic phenotype in pancreatic cancer, we compared expression profiles from 15 primary adenocarcinoma samples with those obtained from 6 distant metastases originated from primary pancreatic adenocarcinoma. Metastatic samples were collected from secondary tumors appearing in different target sites (1 from peritoneum, 1 from ganglion, 4 from liver), from different patients. Using a significance threshold of FDR ≤ 5%, we identified a metastasis-associated signature comprising 355 differentially expressed transcripts (Figure 4). From these, 221 are protein-coding mRNAs and 134 are noncoding RNAs (134/355, 38% of signature), from which 101 map to intronic, 27 to intergenic genomic regions and 6 are known lncRNAs (a complete list is available as Additional file 5, table S3).

Gene enrichment analysis using protein-coding mRNAs differentially expressed in metastatic samples identified the over-representation of genes involved in "nucleic acid transport" and "RNA localization " (*p *< 0.03; *THOC7*, *THOC2*, *RAN*, *NUP85*, *THOC3*, *NUP88*).

A similar analysis performed with gene *loci *harboring intronic lncRNAs differentially expressed in metastasis showed enrichment of gene categories pertaining to "MAPK signaling pathway" (*p *< 0.03; *ARRB1*, *ATF2*, *MAPK1*, *MAP2K5*, *MAP3K1*, *MAP3K14*, *PPP3CB*, *RAPGF2 *and *TGFβR2*), "phosphate metabolic process" (*p *< 0.05; *ABL2*, *ENPP2*, *PTEN*, *CSNK1D*, *TYK2*, *MAPK1*, *MAP2K5*, *MAP3K1*, *MAP3K14*, *PPP3CB*, *PPP2R2A*, *PASK*, *TNK2*, *DAPK1 *and *TGFβR2*), "non-membrane-bounded organelle" (*p *< 0.02; *ABL2*, *GPHN*, *ITPR1*, *SORBS1*, *TYK2*, *MAPK1*, *MAP2K5*, *MAP3K1*, *NDRG1*, *DST*, *MCPH1*, *USH1C*, *MAEA*, *BBS5*, *SLC4A7*, *RAPH1*, *CNN3*, *NR2C2*, *DMD*, *DAZAP1*, *PHF12*, *NOP58*, *ATF3*, *ALMS1*, *STON2*, *DAPK1 *and *MYO5A*) and "actin filament-based process"(*p *< 0.05; *ABL2*, *SORBS1*, *PACSIN2*, *CNN3*, *MYO5A *and *DST*).

Ingenuity Pathway Analysis identified significantly enriched gene networks amongst protein-coding genes differentially expressed in metastasis. The most enriched gene network of differentially expressed protein-coding mRNAs (*p *< 10^-41^) included genes related to "cellular movement, gene expression and immune cell trafficking" (see Additional file 6, Figure S3). Among these we found that up regulation of *S100A4*, *NCAM1 *and *LIMK1 *had already been associated with metastatic behavior in pancreatic cancer [52-54]. While the remaining genes in the network had not been associated with metastasis in pancreatic cancer yet, most of them have previously shown to be involved with malignancy or metastatic behavior in other types of cancer (see Additional file 7, Table S4 for a complete list). Other gene networks enriched in protein-coding mRNAs deregulated in metastatic tumor samples were "cell cycle, genetic disorder, metabolic disease" (21 genes, *p *< 10^-33^), "cardiovascular system development and functions, embryonic development and tissue development" (21 genes, *p *< 10^-30^) and "cancer, tumor morphology and genetic disorder" (19 genes, *p *< 10^-29^). IPA analysis also highlighted the prevalence of genes related to cell death within the metastasis-signature. It comprised 42 protein-coding mRNAs related to apoptosis (19 down-regulated and 23 up-regulated), in line with the notion that perturbation of the normal programmed cell death is involved in the metastatic phenotype in pancreatic cancer [6,55]. Interestingly, we found 6 intronic lncRNAs mapped to *locus *of apoptosis-related genes among those present in the metastasis signature (*ATF2*, *TGFβR2*, *MAP2K5*, *MAP3K1*, *DAPK1 *and *PTEN*).

### Metastasis-associated intronic lncRNAs are expressed with antisense and/or sense orientation relative to corresponding protein-coding genes

**Figure 5:**
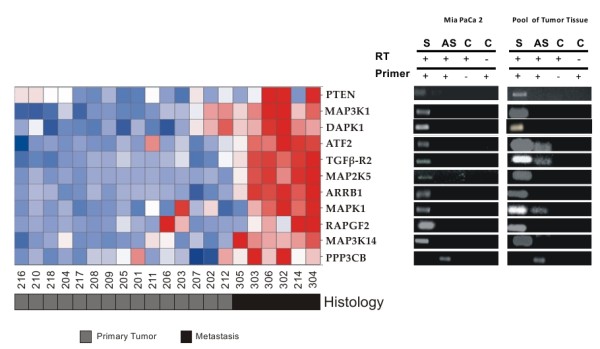
**LncRNAs deregulated in pancreatic cancer metastasis map to intronic regions of genes associated with the MAPK pathway and/or related to apoptosis**. Transcriptional orientation of eleven intronic lncRNAs mapping to MAPK pathway and/or apoptosis-related gene ***loci ***(***ARRB1***, ***ATF2***, ***MAPK1***, ***MAP2K5***, ***MAP3K1***, ***MAP3K14***, ***PPP3CB***, ***RAPGF2***, ***TGFβR2***, ***DAPK1 ***and ***PTEN***) was investigated by strand-oriented reverse transcription followed by PCR. For each gene, sense (S) or antisense (AS) transcription was measured. Controls (C) for the absence of self-annealing during reverse transcription (RT) were obtained by performing RT reactions in the absence of primers (RT+, Primer -). Controls for the absence of genomic DNA contamination were obtained by omitting reverse transcriptase in the RT reaction (RT-, Primer +).

To document in more detail the structure of lnc RNAs mapping intronic regions of gene *loci *related to the MAPK pathway or to apoptosis, which were over-represented in the metastasis-signature, we investigated their orientation relative to the corresponding protein-coding mRNA. Orientation-specific RT-PCR was employed to determine the strandedness of the eleven intronic transcripts mapping to introns of *MAPK *pathway or/and apoptosis-related genes, namely *ARRB1*, *ATF2*, *MAPK1*, *MAP2K5*, *MAP3K1*, *MAP3K14*, *PPP3CB*, *RAPGF2*, *TGFβR2*, *DAPK1 *and *PTEN*. These experiments were performed using total RNA isolated from pancreatic tumor tissue samples or from cultured MIA PaCa-2 cells. Ten transcripts showed evidence of being transcribed with the same (sense) orientation of the corresponding protein-coding mRNA in both pancreatic tissue samples and MIA PaCa-2 cells (Figure 5). Interestingly, a transcript with antisense orientation relative to the protein-coding mRNA was detected in an intronic region of *PPP3CB *in MIA PaCa-2 cells. When RNA from pancreatic tumors was used, antisense intronic transcription was detected in three additional *loci *(*ATF2*, *TGFBR2 *and *MAP3K1*), which produced both sense and antisense messages (Figure 5).

**Figure 6:**
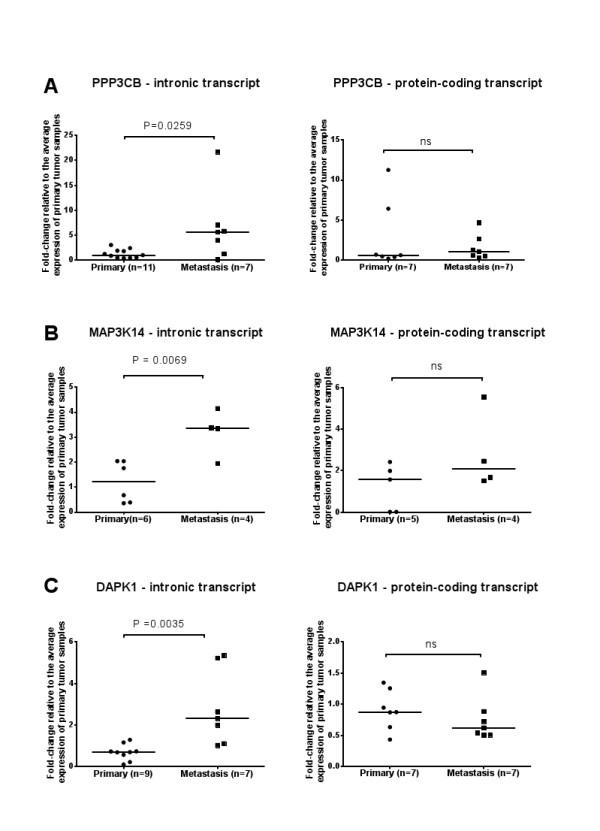
**Expression changes of intronic lncRNAs mapping to *PPP3CB*, *MAP3K14 *and *DAPK1 loci *are not accompanied by changes in the corresponding protein-coding mRNAs**. Relative levels of intronic lncRNAs and protein-coding mRNAs from ***PPP3CB ***(A), ***MAP3K14 ***(B) and ***DAPK1 ***(C) ***loci ***were determined by Real-Time PCR in clinical samples of primary adenocarcinoma (circles) or distant metastasis (squares) from pancreatic cancer patients. For each transcript, the number of tested samples is indicated in the × axis. For each gene, results are expressed as fold-change relative to the average expression of primary tumor samples. For each sample, qPCR assays were performed in triplicate and mean values are shown. House-keeping gene HMBS was used as the endogenous control for normalization across patient samples. All intronic transcripts (left panels) were differentially expressed in metastatic samples at a significance threshold of ***p***< 0.05 (***PPP3CB***, ***p ***= 0.0259; ***MAP3K14***, ***p ***= 0.0069; DAPK1, ***p ***= 0.0035). Protein-coding transcripts (right panels) were not significantly differentially expressed.

The relative abundance of the eleven intronic lncRNAs identified in genes from the *MAPK *pathway or related to apoptosis was evaluated by quantitative Real-Time PCR in RNA samples isolated from primary tumors and distant metastasis. We initially measured the abundance of each of the 11 intronic transcripts in three samples of primary adenocarcinoma and three samples of metastasis. In spite of a great variability due to small sample size, 7 out 11 intronic transcripts showed a similar expression change (same direction) as measured in the microarray. Since the amount of RNA from clinical samples were limiting, we selected for further validation in additional samples three intronic lncRNAs, being one antisense (*PPP3CB*) and two with the same orientation (*MAP3K14 *and *DAPK1*) relative to the protein-coding gene. As shown in Figure 6, statistically significant increased expression of all three intronic lncRNAs was observed.

We next asked if the expression changes of these intronic transcripts would reflect in the expression of the corresponding protein-coding mRNA. Quantitative RT-PCR experiments with primers interrogating protein-coding mRNAs from *PPP3CB, MAP3K14 *and *DAPK1 loci *showed no statistically significant expression change between primary tumors and metastasis samples. To investigate the co-expression of protein-coding mRNAs and intronic lncRNAs from the same *loci *we measured the Pearson correlation of their expression measurements in all samples tested. A high Pearson correlation (r = 0.78, *p *< 0.013) was observed for the *MAP3K14 locus*, suggesting that the intronic sense transcript may be a by-product of pre-mRNA processing of the protein-coding transcript (see discussion for details). No significant correlation between protein-coding mRNAs and intronic lncRNAs was observed for the other two *loci *(*p *> 0.05), leaving open the possibility that intronic RNAs mapping to *PPP3CB *(antisense) and *DAPK1 *(sense) *loci *are noncoding RNAs originated from independent transcriptional events.

## Discussion

In this work we investigated gene expression profiles from clinical samples of pancreatic cancer using a custom cDNA microarray enriched in probes that interrogate long potentially noncoding RNAs mapping to intronic and intergenic regions of the human genome, plus a collection of protein-coding genes previously associated with cancer in the literature. By comparing expression profiles of 38 pancreatic clinical samples with four distinct tissue histologies (primary adenocarcinoma, adjacent non-tumor tissue, chronic pancreatitis, metastasis), we detected in all types of pancreatic tissues studied a proportion of intronic and intergenic transcripts comparable to the one observed for protein-coding mRNAs. There are several reports of aberrant expression of microRNAs [21-24], but this is to our knowledge the first time that the expression of lncRNAs has been studied in pancreatic cancer.

We observed that most intronic and intergenic transcripts expressed in pancreatic tissues have little or no coding potential (96% of total). Comparison with sequence contigs resulting from the assembly of EST/mRNA data produced in our group [26] showed that these transcripts have a mean size of at least 779 nt, being longer than the EST probes deposited in the microarrays, which represent indeed only parts of longer noncoding RNAs transcribed from intronic regions. Most putative intergenic transcripts (~81%) were located more than 1 kb apart from an UTR of an annotated gene, suggesting that for the most part, these are indeed intergenic transcripts rather than uncharacterized untranslated regions of incomplete mRNAs.

While it is clear that lncRNAs may exert diverse cellular functions through multiple molecular mechanisms [12,56,57], it has been suggested that a fraction of the transcriptome noncoding complement may correspond to transcriptional noise resulting from RNA polymerase activity in regions of open chromatin or intronic segments of processed mRNAs [58]. Our expression measurements of intronic lncRNAs do not permit to distinguish between i) intron lariats resulting from splicing of a pre-mRNA or ii) independent transcriptional units located within intron-annotated genomic regions. We have focused on poly(A+)-selected RNA fractions followed by oligo-dT primed reverse transcription to minimize the chance of labeling targets from non-polyadenylated spliced lariats. We argue that the identification of subsets of transcripts that map to intronic regions and whose steady-state levels allows the detection by microarrays indicate that these are not rapidly turned-over intron lariats. We have also performed a series of analysis to obtain additional evidence to support the notion that intronic/intergenic lncRNAs detected in pancreatic tissues are indeed *bona fide *cellular transcripts, as discussed below.

We first sought independent confirmation of intronic/intergenic lncRNA expression using RNAseq data generated from 9 distinct tissue libraries [30]. We found that approximately 80% of intronic/intergenic lncRNAs detected in pancreatic tissues were also detected in at least one RNAseq library (Figure 1). Most transcripts confirmed by the RNAseq data were detected i) only in a single tissue type other than pancreas, or ii) in all 9 tissue libraries plus pancreas, indicating the prevalence of subsets of noncoding transcripts with broad or specific tissue-type expression patterns, respectively (Figure 1).

While only a fraction of the intronic/intergenic lncRNAs expressed in pancreatic tissues overlapped evolutionarily conserved DNA elements in vertebrates, mammals and primates, we observed a significant enrichment (p < 0.05) compared to randomly selected control regions. This result suggests that at least a fraction of these lncRNAs are under purifying selection in the vertebrate lineage and therefore must be biologically functional. For the remaining transcripts, absence of sequence conservation should not be taken as evidence of no biological relevance, since it is known that well-characterized functional lncRNAs are poorly conserved across their global sequence [59].

As proposed by Washielt et al. [60], mapping conserved RNA secondary structure may lead to the discovery of novel functional lncRNAs. We found that a small fraction of lncRNAs expressed in pancreas (15%. i.e. 49/335) are predicted to form stable structural domains that could be important for their processing or biological function. It is well documented in the literature that small regulatory RNAs can be generated by processing of long RNA precursors transcribed from intronic and intergenic regions of the genome [56]. To ask what fraction of our set of lncRNAs expressed in pancreatic tissues could be precursor of small RNAs we compared their sequences to those of known microRNA and snoRNA [32,33]. Only a discrete overlap was found, indicating that long intronic/intergenic transcripts are predominantly not precursors of known microRNAs/snoRNAs, yet leaving open the possibility that these transcripts may represent precursors of uncharacterized novel small RNAs.

We found significant enrichment of H3K4me3, a promoter-associated chromatin mark frequently found in RNA Pol II transcribed regions [35,61], in the vicinity (up to 2 kb) of intronic (p < 0.05) noncoding transcripts as compared to randomly selected genomic DNA sequences. A comparable H3K4me3 enrichment was observed nearby known protein-coding transcripts, suggesting that transcription of protein-coding mRNAs and intronic lncRNAs initiates at promoter regions with similar chromatin contexts. We also observed a significant enrichment of CAGE tags proximal to known start sites of intronic lncRNAs expressed in pancreatic tissues, corroborating the notion that at least a fraction of these is independent transcriptional units. Since pancreatic tissues were absent from the study that generated the CAGE tags used for cross-reference, these results possibly underestimate the co-localization of intronic/intergenic lncRNAs with *bona fide *transcription start sites of capped transcripts.

Differently from protein-coding mRNAs, we did not find significant enrichment of CpG island in the vicinity of intronic and intergenic RNA sequences expressed in pancreatic tissues. Based on this observation, we propose that methylation of CpG islands is not involved in the transcriptional regulation of most intronic/intergenic lncRNAs expressed in pancreatic tissues. Nonetheless, the full set of observations regarding the structure, conservation and genomic context argues that at least a fraction of intronic/intergenic transcripts detected in pancreatic tissues are independent transcriptional units rather than transcriptional noise originated from random Pol II firing [62], prompting us to investigate in more detail their relative expression levels in tumor and non-tumor pancreatic tissues.

Differential expression of intronic lncRNAs in prostate and renal cancer has already been documented [17,28]. Here we extend these observations to pancreatic cancer, asking whether there were sets of intronic/intergenic lncRNAs deregulated in clinical samples of pancreatic tumor. Comparing expression profiles from primary tumors with samples from histologically non-malignant pancreatic tissue and chronic pancreatitis (CP) we identified a 147-gene signature correlated with primary pancreatic tumor. This strategy was devised to favor the identification of tumor specific markers rather than transcripts associated with the stromal cell component, which is augmented in both tumor and CP samples [36,37]. We sought to validate the pancreatic cancer expression signature by performing a meta-analysis with published gene expression studies of pancreatic cancer. Only 23% of the protein-coding mRNAs present in our pancreatic cancer signature were also identified in other reports. This modest overlap can be accounted for by differences in platforms and the heterogeneity of pancreatic tumor samples. Notwithstanding, we observed a high agreement (17/24, 71%) between the expression changes measured in our signature and those retrieved from published data, which provides independent support for our result and validates our sample set and methodological approach. This set included genes already reported in the literature as differentially expressed in pancreatic cancer and that have been investigated as biomarkers for pancreatic cancer (i.e. *S100A6 *[47], *S100P *[46], *TIMP1 *[63] and *NF-κB *[64]). In agreement with previous findings [5], the analysis of gene enriched categories in the pancreatic cancer expression signature indicated the over-representation of genes involved in focal adhesion. Over-representation of focal adhesion genes in the pancreatic cancer signature is suggestive that deregulation of genes encoding proteins involved in the connection and signaling to the extracellular matrix plays an important role in the malignant transformation and/or maintenance of pancreatic adenocarcinomas. This set included integrin beta 5 (*ITGB5*), which we found to be upregulated in pancreatic adenocarcinoma. Itgb5 protein has been investigated as diagnostic biomarker in non-small cell lung cancer [65] and is target of the inhibitor drug EMD121974, which is under clinical trial [66]. Thus, *ITGB5 *is an attractive candidate to be tested as biomarker and/or new drug target in pancreatic cancer.

Interestingly, a significant fraction (29%) of the 147-gene signature correlated with primary pancreatic tumor was comprised by lncRNAs mapping to intronic or intergenic regions, suggesting that noncoding RNAs could exert roles related to tumorigenesis of pancreatic cancer. This result prompted us to investigate the existence of subsets of lncRNAs with expression levels altered in metastatic samples.

We identified a statistically significant metastasis signature of 355 differentially expressed transcripts that includes 220 protein-coding genes, 134 intronic/intergenic transcripts and 6 known lncRNAs (Figure 4 and Additional file 5, Table S3). In addition to protein-coding genes previously shown to be deregulated in pancreatic metastasis (7 out of 19), the metastasis signature comprises known genes already associated to metastasis in other types of cancer (Additional file 7, Table S4), thus pointing to potentially interesting candidates for testing as new targets for treatment of the metastatic disease in pancreatic cancer.

The significant fraction of lncRNAs in the metastasis signature (38% of total) suggests that deregulation of these lncRNAs could also be associated with the metastatic process. Expression changes of protein-coding mRNAs from genes of the MAPK pathway has already been described in pancreatic carcinoma [67-69]. Here we found 9 intronic lncRNAs mapped to genes correlated to the MAPK pathway in the metastasis signature. We also identified expression changes in gene *loci *related to apoptosis, including 42 protein-coding mRNAs and 6 intronic lncRNAs; this pathway was one out of 12 described by Jones *et al. *[6] as genetically altered in pancreatic cancer. Four intronic lncRNAs belong to both categories. These results prompted us to document in more detail the nature of the 11 transcripts mapping to intronic regions of gene *loci *associated with the MAPK pathway or related to apoptosis, i.e., their relative orientation to the corresponding protein-coding mRNAs.

Strand-specific RT-PCR assays using RNA aliquots from tumor tissue samples showed that 4 intronic transcripts have antisense orientation relative to the protein-coding mRNA: *PPP3CB*, *ATF2*, *TGFBR2 *and *MAPK1*. Antisense transcripts originated in *PPP3CB *intronic regions were also detected in MIA PaCa-2 cells. The antisense orientation relative to the corresponding protein-coding mRNA provide strong evidence to support that these noncoding RNAs are produced from independent transcriptional units, possibly under control of a different promoter region.

Transcripts mapping to intronic regions with the same orientation of the corresponding protein-coding mRNA were detected in the *ATF2*, *TGFBR2 *and *MAPK1*, as well as in the 7 other gene *loci *tested (*ARRB1*, *MAP3K1*, *MAP3K14*, *MAP2K5*, *PTEN, DAPK1 *and *RAPGF2*), in both tissue and MIA PaCa-2 RNA samples. These sense-oriented intronic transcripts could indeed be *bona fide *RNAs originated from independent transcription, but also result from reverse transcription of unprocessed mRNA precursors or of stable RNA lariats generate during pre-mRNA splicing. Further experiments will be necessary to determine the precise nature of these sense-oriented intronic RNAs.

The relative abundance of two sense (*DAPK1, MAP3K14*) and one antisense-oriented (*PPP3CB*) intronic transcripts in samples of primary pancreatic adenocarcinoma and pancreatic metastases was independently accessed by qRT-PCR, confirming the results measured in the microarray hybridizations. Four additional intronic lncRNAs showed concordant results between qRT-PCR and the microarrays (*ARRB*, *RAPGF2*, *ATF2 *and *PTEN*). Expression changes of 4 intronic lncRNAs were not concordant between qRT-PCR and microarray (*MAP3K1*, *TGFBR2*, *MAP2K5 *and *MAPK1*). The amount of RNA and the number of patient tissue samples available for the qRT-PCR experiments were limiting, and the marginally significant and non-validated lncRNA candidates were tested only in few samples in an initial round of validation. It is possible that some of the intronic lncRNA candidates that failed the initial round of validation would still be validated as differentially expressed if tested in additional tissue samples. However, an alternative explanation for the non-validation of some candidates is the presence of array hybridization artifacts such as cross-hybridization or target amplification biases.

Intragenic lncRNAs have been shown to modulate in *cis *the expression of mRNAs expressed in the same *locus *[29,70,71]. We measured the relative abundance of mRNAs produced in the *PPP3CB*, *DAPK1 *and *MAP3K1*4 *loci *in the same samples and did not observe statistically significant expression differences between primary tumors and metastasis. This result indicates that intronic RNAs produced in these *loci *do not affect in *cis *the abundance of the corresponding protein-coding transcripts. This conclusion is also supported by the absence of significant correlation between expression levels of protein-coding and noncoding RNAs originating from *PPP3CB *and *DAPK1 loci*. The possibility that intronic lncRNAs differentially expressed in metastatic samples may exert regulatory functions acting in *trans *is compelling and warrants further studies.

It has been shown that a significant portion of the noncoding component of the human transcriptome is comprised of non-polyadenylated RNAs [10]. We note that our analysis was limited to the set of lncRNAs interrogated by the array platform (Table 1) and by the use of poly(A+)-enriched RNA, and therefore is not comprehensive in terms of describing the full complement of lncRNAs expressed in pancreatic tissues. Thus, additional studies using unbiased approaches such as RNAseq or tiling arrays will be required to catalog all poly(A+) and poly(A-) transcripts expressed in pancreatic tissues with distinct degrees of malignancy and for the identification of novel regulatory lncRNA candidates involved in the malignant transformation and tumor progression.

## Conclusions

In this work we report that noncoding RNAs originating from intronic and intergenic genomic regions are expressed in tumor and non-tumor pancreatic tissues. Enrichment of promoter-associated chromatin marks plus the observation of antisense orientation of intronic transcripts relative to mRNAs expressed from the same *loci *provide evidence that these messages are not by-products of random transcription or pre-mRNA splicing but rather, are independent transcriptional units. Further investigation will be required to determine the biogenesis of these lncRNAs.

We identified gene expression signatures correlated to primary and metastatic stages of pancreatic cancer, which in addition to protein-coding mRNAs comprise collections of long intronic and intergenic noncoding RNAs. Further studies will be necessary to reveal possible biological functions and molecular mechanisms exerted by these lncRNAs in tumorigenesis and/or progression of pancreatic tumors.

In summary, our work contributes with novel candidate biomarkers of pancreatic cancer and highlights the importance of investigating the biological relevance of long noncoding RNAs in order to fully understand the molecular basis of the disease.

## Methods

### Patient Samples and Cell Lines

A total of 38 pancreatic samples stored in freshly-frozen tissue collections were obtained with informed consent from patients seen at Hospital das Clínicas, Faculdade de Medicina da Universidade de São Paulo (HC-FMUSP). Primary tumor tissues (T) were obtained from 15 patients with no evidence of metastasis. Nine samples of histologically normal pancreatic tissue fragments (NT) were dissected from non-neoplasic tissue sections adjacent to tumor sites. Six samples of metastases originated from primary pancreatic tumors (M) were obtained from biopsies in affected organs (one from peritoneum, one from ganglion and four from liver tissues). Eight tissue samples from patients with chronic pancreatitis (CP) were also collected. All tissue sections were reviewed by a pathologist for histological confirmation and whenever necessary, macro-dissected to guarantee that 80% or more of the sections used for gene expression analysis were composed of neoplastic/pancreatitis tissue.

Pancreatic carcinoma cell lines MIA PaCa-2 were obtained from the American Type Culture Collection and maintained using DEMEM supplemented with 10% (v/v) fetal calf serum (FCS), 3 mM L-glutamine, 100 μg/ml streptomycin and 100 U/ml penicillin.

### RNA extraction and microarray target preparation

Total RNA was extracted from pancreatic tissue samples (50-100 mg tissue) using Trizol (Invitrogen) according to manufacturer's recommendations. RNA cleanup including a DNase I digestion step was performed using RNeasy spin columns (Qiagen). RNA integrity was measured by the relative abundance of 28S/18S ribosomal subunits, verified through micro fluid capillary electrophoresis (Agilent Bioanalyzer 2100).

To generate cRNA targets, 1 μg of total RNA from each sample was linearly amplified in two rounds of reverse transcription followed by *in vitro *transcription according to Wang et al. [72]. Briefly, oligo dT-T7 was used to prime first-strand cDNA synthesis (SuperScript III First Strand Synthesis - Invitrogen). After second-strand cDNA synthesis (cDNA Polymerase Mix - Clontech), cRNA targets were produced by *in vitro *transcription (MegaScript T7 - Ambion). In the second round of amplification, cRNAs produced in the first-round were reverse transcribed using random hexamer primers and used as template for *in vitro *transcription in the presence of amino-allyl UTP. Prior to hybridization, cRNA targets were labeled by coupling with mono-reactive Cy5-esters (Amersham). Quantification of cDNA yield and dye incorporation was performed using a NanoDrop spectrophotometer (Thermo Scientific). Typically, 50-100 μg of cRNA were obtained following two rounds of linear amplification. Two sets of cRNA targets were generated from each RNA sample and independently hybridized to microarray slides.

### Microarray design and hybridization

Construction of the spotted custom-cDNA microarray platform was described previously [17]. Probes were selected from the over 1 million EST clone collection generated during the Human Cancer Genome Project, a large-scale EST sequencing project that used cDNA libraries generated from poly(A) mRNA derived from over 20 different types of human tumors [73,74]. Transcripts from the EST dataset were annotated as protein-coding, putative intronic lncRNA or intergenic lncRNA following mapping to the human genome sequence and cross-referencing with genome mapping coordinates of annotated genes (RefSeq dataset) [17]. Intronic/intergenic lncRNAs used for microarray spotting were randomly selected from the annotated EST dataset. Transcripts annotated as "intronic lncRNAs" comprise sequences that mapped within an intronic region of a protein-coding gene. Transcripts annotated as "intergenic lncRNAs" comprise sequences that map to genomic regions devoid of any annotated gene. To be annotated as intronic or intergenic a given transcript could not overlap a genomic region spanning an exon of annotated protein-coding genes. "Known lncRNAs" refer to transcripts whose genomic coordinates overlap fully with the coordinates of noncoding RNAs from the RefSeq dataset (accession Id = NR_nnnnnn). Transcripts annotated as "protein-coding gene" overlapped with exons of protein-coding RefSeq transcripts in genomic space. To account for possible unannotated intron retention events, partial transcripts mapping to exon/intron boundaries were annotated as "exonic".

In the course of this work microarray probes were re-mapped to the latest version of the human genome (hg19) and re-annotated to reflect updated RefSeq and UCSC gene models (Oct. 2010). Each glass-slide contains 3,355 cDNA fragments spotted in duplicate, plus positive (cDNA from housekeeping genes) and negative (plant and bacterial DNA) controls. Spotted cDNAs comprise 722 ESTs mapping to intronic regions of well-annotated (RefSeq) protein-coding genes, 74 ESTs mapping to known RefSeq lncRNAs, 188 ESTs mapping to intergenic regions of the genome. The array also contained 2,371 ESTs mapping to exons of protein-coding genes associated with cancer based on a literature search [17], comprising genes involved in apoptosis, tumorigenesis, metastasis, cancer metabolism and cancer progression.

For each sample, cRNA targets were ressuspended in a final volume of 200 μl of 1× Microarray Hybridization Solution v.2.0 (GE Healthcare) containing 25% formamide, denaturated at 92°C for 2 minutes and incubated with microarrays at 42°C for 16 hours using an automated slide processor (GE Healthcare). Following sequential washes in 1× SSC; 0.2% SDS, 0.1× SSC; 0.2% SDS and 0.1× SSC; 0.2% SDS, microarray slides were scanned immediately in a Generation III Microarray Systems Scanner (Molecular Dynamics/GE Healthcare). For each sample, two slides were hybridized with different preparations of cRNA targets. As probes are spotted in duplicate in the arrays, a total of 4 replicate measurements were collected for each cDNA for each sample.

### Data processing and analysis

Cy5-intensity measurements from hybridized targets were extracted from array images using the ArrayVision software (Imaging Research Inc.). To make the expression values comparable across all samples tested, the raw data was normalized by the quantile method [75]. Next, for each slide, the fifty percent of probes with the lowest intensity values were filtered out. A mean expression value for each probe, in each patient sample, was calculated when at least 3 out of 4 replicates showed valid measurements. Only probes with valid measurements in at least 75% of the samples in any of the histological groups (NT, T, PA or M) were selected, resulting in a total of 1,607 probes for further analysis. The ComBat program was used to remove systematic variations in gene expression across experiments resulting from the use of different batches of microarrays [76]. Inter-slide Pearson correlations using normalized intensities from all probes in the array were calculated before and after filtering and normalization of data intensities. Raw data intensities showed average inter-slide correlations of 0.63, whereas normalized data showed inter-slide correlation of 0.83.

Raw and normalized microarray intensities were deposited in the Gene Expression Omnibus database (GEO - http://www.ncbi.nlm.nih.gov/geo/) under accession number GSE30134.

Significance analysis of microarrays (SAM) approach [77] was employed to identify gene expression signatures correlated to tissue histology, with the following parameters: two or multi-class response, 1000 permutations, K-Nearest Neighbors Imputer, and false discovery rate (FDR) ≤ 10% or ≤5%. For representation of gene expression measurements in heat-maps, samples were ordered according to their individual correlation to the average profile of the primary tumor samples.

### Bioinformatics analyses

We used the BEDTools software package [78] to cross-reference genome mapping coordinates (GRCh37 build, hg19) of our dataset of intronic/intergenic noncoding sequences with those from the various datasets used in this analysis and available through the UCSC Genome Browser [66]: i) RNAseq data of PolyA^+ ^RNA-derived libraries from 9 tissues [30], ii) RIKEN CAGE tag data from PolyA^+ ^RNA-derived libraries from 6 cell lineages [79]; iii) ChIP-seq data of H3K4m3 DNA binding sites [35], iv) conserved DNA elements in vertebrates, mammalian and primates calculated with PhastCons program [66], v) predicted CpG islands [80] and vi) intronic noncoding RNAs assembled from EST/mRNA GenBank data [26].

To test the statistical significance of the overlap between our dataset of intronic/intergenic lncRNAs and the datasets of conserved elements and regulatory motifs (H3K4me3, CpG islands, CAGE tags), we generated 100 groups of randomly selected sequences from intronic or intergenic regions of the human genome matching in number, length and CG content our set of expressed noncoding sequences. As pre-processing of CAGE tag data, coordinates of overlapped tags were clustered and only clusters containing at least 5 tags were considered for further analysis. Fischer's exact test was used to test the statistical significance (*p *< 0.05 threshold) of the enrichment of conserved DNA elements in intronic/intergenic lncRNAs relative to the enrichment observed for the 100 random sequence sets.

For the analysis of transcription regulatory elements, we first computed the distance of the closest H3K4me3 marks, predicted CpG islands and CAGE tags to our set of expressed intronic/intergenic lncRNAs, expressed protein-coding mRNAs, and of the set of 100 random groups. Transcription regulatory elements mapping to 5'UTRs of known transcripts (RefSeq and UCSC genes) were removed to avoid the contribution of signals at start sites of known genes to the enrichment of regulatory elements at start sites of intronic lncRNAs mapping nearby.

Only regulatory elements distant up to 10 kb of sequence boundaries were considered. Next, non-parametric Kolmogorov-Smirnov (KS) test statistics, implemented using the Deducer package under R language [81] was used to compare the distance distributions of H3K4me3 marks, predicted CpG islands and CAGE tags observed for intronic or intergenic noncoding sequences and those calculated for each of the 100 control random sets. Distance distributions of regulatory motifs from intronic/intergenic lncRNAs/protein-coding mRNAs were considered significantly different from the obtained by chance if all KS *p-values *calculated using each random set were smaller than 0.05.

Protein-coding potential of intronic and intergenic lncRNAs was evaluated using the Coding Potential Calculator software [31] with default parameters. RNAz program [34] was used to predict structurally conserved and thermodynamically stable RNA secondary structures. Only predicted structures with P > 0.5 were considered as containing conserved secondary structures.

### Orientation-specific RT-PCR

Aliquots of DNAse-treated total RNA from a pool of six pancreatic tumor tissues samples or from Mia PaCa-2 cells were used as template in orientation-specific reverse transcription reactions. Reactions were performed with 200 ng total RNA plus 2.5 μM of oligonucleotide primers designed to detect sense or antisense strand intronic transcripts, relative to the orientation of the mRNA from the same *locus*. SuperScript III™ Super Mix (Invitrogen) was used according manufacture's recommendations, with the following modification: reverse transcription reaction was increased to 57°C to limit RNA self-annealing. To verify the absence of priming due to self-annealing or genomic DNA contamination, control reactions were performed without addition of primers or of reverse transcriptase, respectively.

### Real-time RT-PCR

One microgram aliquots of DNase-treated total RNA from 11 clinical samples of primary pancreatic tumors (5 already used in the microarray experiments and 6 new samples) and 6 of distant metastases with pancreatic origin (4 already used in the microarray and 2 new samples) were reverse transcribed using SuperScript III™ Super Mix kit (Invitrogen) and random hexamer primers according to manufacturer's recommendation. Relative abundance of selected transcripts primary tumor/metastasis samples was determined by real-time PCR using the ABI PRISM^® ^7300 Real Time PCR System and the SYBR Green PCR Master Mix kit (Applied Biosystems). Reactions were performed in a final volume of 20 μl containing a 5 μl aliquot of diluted cDNA (1:7) and 1 μM of forward and reverse gene-specific primers. Expression levels of hydroxymethylbilane synthase (HMBS) [82] appeared to be constant and was used as a reference gene to make expression measurements comparable across all different samples tested. For quantitative results, the level of each transcript was normalized by the level of HBMS, and represented as fold change using the 2^-ΔΔCt ^method [83], where ΔΔCt = (Ct candidate gene in sample × - Ct reference gene in sample X)_sample _- mean ΔC_t _of all primary tumor samples tested.

## Competing interests

The authors declare that they have no competing interests.

## Authors' contributions

Conceived and designed the experiments: EMR, MCCM, MSK, ACT. Performed the experiments: ACT, MFF, BD,VMC and RSF. Analyzed the data: ACT, SVA, EMR. Wrote the paper: ACT, EMR. All authors read and approved the manuscript.

## Supplementary Material

Additional File 1 **Figure S1: Intronic/intergenic lncRNA are enriched in conserved DNA elements**. Genomic coordinates of conserved DNA elements identified in Vertebrate, Placental or Primate sequences (see *Methods *for details) were cross-referenced to those from lncRNAs expressed in pancreatic tissue (blue bars). These presented a higher overlap with evolutionary conserved DNA elements than the observed for a random set of genomic DNA sequences with same length and CG content (gray bars) (Fisher's test *p <*0.05). Bar heights indicate the number of lncRNAs that overlap conserved DNA elements in each taxonomic group divided by the total number of conserved DNA elements present in each group. Click here for file

Additional File 2 **Table S1: List of transcripts differentially expressed in PDAC relative to chronic pancreatitis and non tumor tissue samples combined**. Click here for file

Additional File 3 **Table S2: Validation of protein-coding genes differentially expressed in PDAC by meta-analysis of published data**. Click here for file

Additional File 4 **Figure S2: Genes modulated in pancreatic cancer are involved in cellular movement, cell-to-cell signaling interactions and endocrine system**. Ingenuity Pathway Analysis was used to identify gene networks over-represented among the set of 104 protein-coding genes differentially expressed in PDAC samples. The most significantly enriched network (*p *= 10^-43^) is comprised by 23 differentially expressed genes measured in the microarrays. Red indicates higher expression, and green, lower expression in tumor tissues relative to chronic pancreatitis and adjacent non-tumor tissue samples combined. Click here for file

Additional File 5 **Table S3: List of genes differentially expressed between PDAC and metastatic tissue samples**. Click here for file

Additional File 6 **Figure S3: Genes modulated in metastatic pancreatic cancer are involved in cellular movement, gene expression and immune cell trafficking**. Ingenuity Pathway Analysis was used to identify gene networks over-represented among the set of 221 protein-coding genes differentially expressed in PDAC relative to metastasis tissue samples. The most significantly enriched network (*p*= 10^-41^) is comprised by 24 differentially expressed genes measured in the microarrays. Red indicates higher expression, and green, lower expression in metastasis relative to PDAC tissue samples. Click here for file

Additional File 7 **Table S4: Genes from the pancreatic cancer metastasis signature related to tumor aggressiveness in other cancer types**. Click here for file
