# Supplementary figures and images for: Long noncoding intronic RNAs are differentially expressed in primary and metastatic pancreatic cancer
Source: Mol Cancer. 2011 Nov 13;10:141. doi: 10.1186/1476-4598-10-141 (PMC3225313; doi:10.1186/1476-4598-10-141)

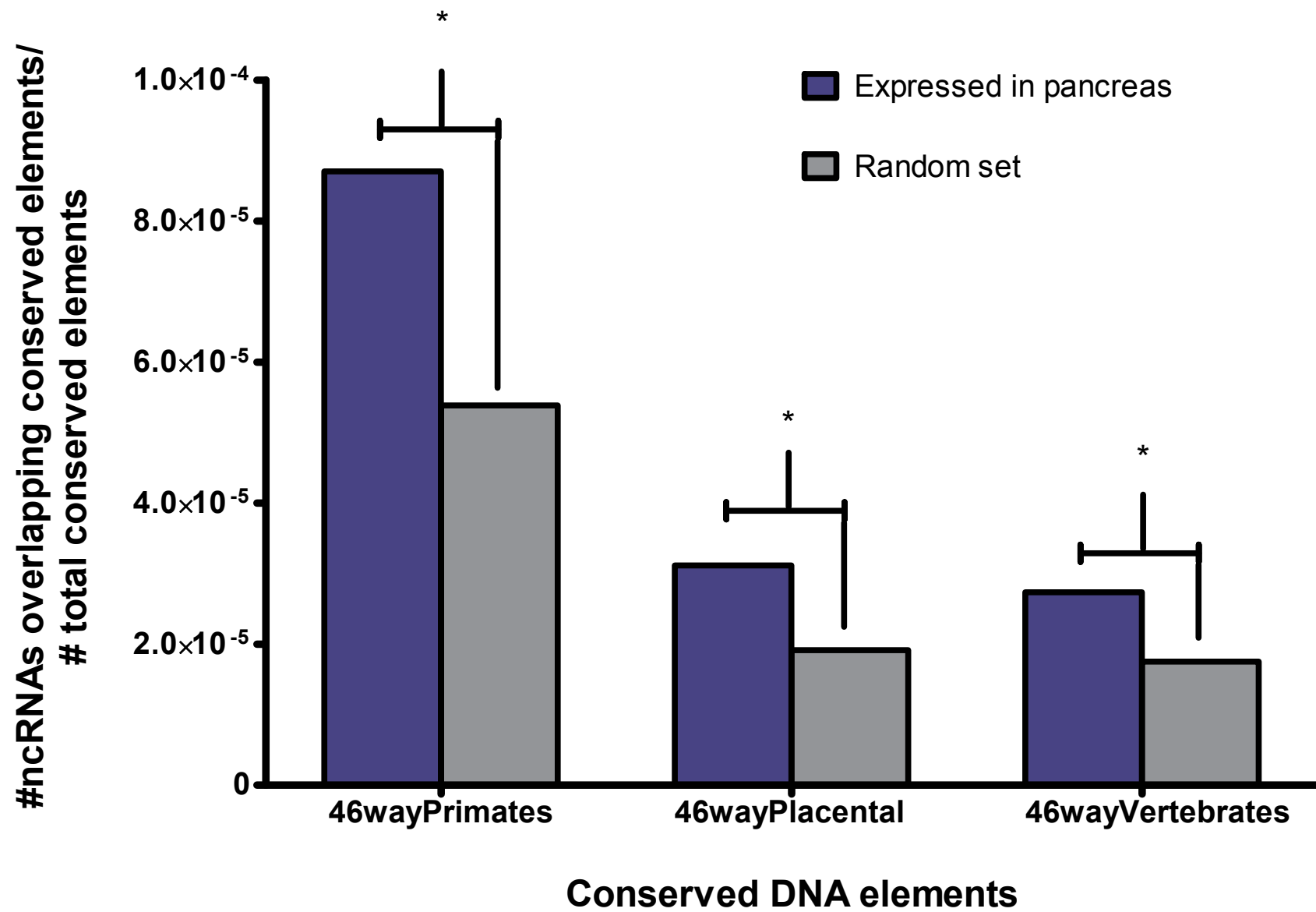

Supplement: Additional File 1 — Figure S1: Intronic/intergenic lncRNA are enriched in conserved DNA elements. Genomic coordinates of conserved DNA elements identified in Vertebrate, Placental or Primate sequences (see Methods for details) were cross-referenced to those from lncRNAs expressed in pancreatic tissue (blue bars). These presented a higher overlap with evolutionary conserved DNA elements than the observed for a random set of genomic DNA sequences with same length and CG content (gray bars) (Fisher's test p <0.05). Bar heights indicate the number of lncRNAs that overlap conserved DNA elements in each taxonomic group divided by the total number of conserved DNA elements present in each group. [file 1476-4598-10-141-S1.PDF]

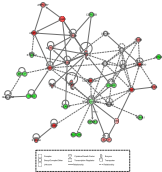

Supplement: Additional File 4 — Figure S2: Genes modulated in pancreatic cancer are involved in cellular movement, cell-to-cell signaling interactions and endocrine system. Ingenuity Pathway Analysis was used to identify gene networks over-represented among the set of 104 protein-coding genes differentially expressed in PDAC samples. The most significantly enriched network (p = 10-43) is comprised by 23 differentially expressed genes measured in the microarrays. Red indicates higher expression, and green, lower expression in tumor tissues relative to chronic pancreatitis and adjacent non-tumor tissue samples combined. [file 1476-4598-10-141-S4.PDF]

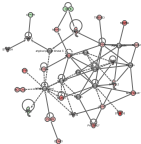

|                                                                                 |                                                                                   |                                                                                   |                                                                                   |                                                                                   |
|---------------------------------------------------------------------------------|-----------------------------------------------------------------------------------|-----------------------------------------------------------------------------------|-----------------------------------------------------------------------------------|-----------------------------------------------------------------------------------|
| 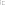 | 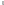 | 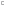 | 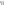 | 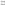 |
| Green                                                                           | Red                                                                               | Black                                                                             | Triple                                                                            | Coordination                                                                      |
| Atom                                                                            | Atom                                                                              | Atom                                                                              | Bond                                                                              | Bond                                                                              |

Supplement: Additional File 6 — Figure S3: Genes modulated in metastatic pancreatic cancer are involved in cellular movement, gene expression and immune cell trafficking. Ingenuity Pathway Analysis was used to identify gene networks over-represented among the set of 221 protein-coding genes differentially expressed in PDAC relative to metastasis tissue samples. The most significantly enriched network (p= 10-41) is comprised by 24 differentially expressed genes measured in the microarrays. Red indicates higher expression, and green, lower expression in metastasis relative to PDAC tissue samples. [file 1476-4598-10-141-S6.PDF]
